# Effect of Lycopene on the Growth Performance, Antioxidant Enzyme Activity, and Expression of Gene in the *Keap1-Nrf2* Signaling Pathway of Arbor Acres Broilers

**DOI:** 10.3389/fvets.2022.833346

**Published:** 2022-03-14

**Authors:** Sibo Wang, Hongzhi Wu, Yunhui Zhu, Hongxia Cui, Ji Yang, Mingyuan Lu, Huangzuo Cheng, Lihong Gu, Tieshan Xu, Li Xu

**Affiliations:** ^1^College of Animal Science and Technology, Northeast Agricultural University, Harbin, China; ^2^Tropical Crop Genetic Resource Research Institute, Chinese Academy of Tropical Agricultural Sciences, Haikou, China; ^3^Inner Mongolia Ordos City Agricultural and Forestry Technology Extension Center, Ordos, China; ^4^Institute of Animal Science & Veterinary, Hainan Academy of Agricultural Science, Haikou, China

**Keywords:** lycopene, growth performance, antioxidant enzyme activity, *Keap1-Nrf2*, broilers

## Abstract

The objective of this study was to determine the effect of dietary lycopene supplementation on the growth performance, antioxidant enzyme activity of serum and liver, and gene expressions associated with Kelch-like ech-associated protein-1 (*Keap1*)/Nuclear Factor E2-related factor 2 (*Nrf2*) pathway in liver of Arbor Acres broilers. A total of 288 1-day-old male broilers were randomly divided into 4 treatments with 6 replicates and 12 chickens for each replicate. The control group was fed with the basal diet, while the treated groups were fed with the basal diet with 10, 20, and 30 mg/kg lycopene in powder. Feed and water were provided *ad libitum* for 42 days. Compared with the control group, (a) the average daily gain increased (*p* = 0.002 vs. *p* = 0.001) and the feed conversion ratio decreased (*p* = 0.017 vs. *p* = 0.023) in groups treated with lycopene in the grower and whole phases, and the average daily feed intake was quadratically affected (*p* = 0.043) by lycopene in the grower phase; (b) the serum superoxide dismutase content was linearly affected (*p* = 0.035) by lycopene at 21 days; (c) the serum glutathione peroxidase content, superoxide dismutase content, and total antioxidant capability were higher (*p* = 0.014, *p* = 0.003, and *p* = 0.016, respectively) in the 30 mg/kg lycopene group at 42 days; (d) the liver glutathione peroxidase and superoxide dismutase contents in groups treated with lycopene were higher (*p* ≤ 0.001 vs. *p* ≤ 0.001) at 21 days; (e) the liver glutathione peroxidase content was higher (*p* ≤ 0.001) in the 20 and 30 mg/kg lycopene groups, at 42 days; (f) the mRNA expression levels of *Nrf2, superoxide dismutase 2, NAD(P)H quinone dehydrogenase 1*, and *heme oxygenase 1* genes were higher (21 days: *p* = 0.042, *p* = 0.021, *p* = 0.035, and *p* = 0.043, respectively; 42 days: *p* = 0.038, *p* = 0.025, *p* = 0.034, and *p* = 0.043, respectively) in the 20 and 30 mg/kg lycopene groups at 21 and 42 days. The 30 mg/kg lycopene concentration improved the growth performance, antioxidant enzyme activity in serum and liver, and gene expression in the *Keap1-Nrf2* signaling pathway of Arbor Acres broilers.

## Introduction

Oxidative stress can be caused by moldy feed, poor feeding environment, and imbalance of intestinal flora in poultry (1–3). The phytochemicals, such as oleanolic acid, polyphenols, and carotenoids, could alleviate the adverse effects of the reactive oxygen species in animals (4–6). Lycopene is a polyunsaturated aliphatic hydrocarbon with 11 conjugated double bonds and two non-conjugated double bonds in its chemical structure (7, 8). It is an acyclic isomer of β-carotene that mainly exists in ripe tomatoes and is the most potent antioxidant among most carotenoids (9, 10). As an additive, lycopene is recognized as a class A nutrient by the Food Additives Committee, the Food and Agriculture Organization of the United Nations, and the World Health Organization for its antioxidant and coloring functions (11). In the antioxidant defense network of the poultry, the key antioxidant enzyme system is mainly established by superoxide dismutase, glutathione peroxidase, and catalase (12, 13). It was found that adding dried tomato pomace to the diet could improve the activities of superoxide dismutase and catalase in the plasma of the Lohman Layer (14), the laying rate increased, and malondialdehyde content in serum and eggs decreased (15), indicating that the lycopene has an antioxidant function, but few studies about the antioxidant mechanisms of lycopene are found. The Kelch-like ech-associated protein-1/Nuclear Factor E2-related factor 2 (*Keap1-Nrf2*) signaling pathway regulates the transcription of antioxidant genes, which is considered to be the most critical pathway in the cellular antioxidant mechanism (16, 17). Wang et al. (18) found that the lycopene degraded intracellular *Keap1* and activated *Nrf2* could prevent cutaneous tumors. Moreover, it was also found that inhibiting *Keap1* expression and enhancing *Nrf2* expression in broiler muscles can relieve stress caused by environment (19), and activating *Nrf2*/heme oxygenase 1 (*HO-1*) pathway can ameliorate oxidative stress in the aging chicken ovary (7). It is hypothesized that the lycopene improves antioxidative potentials by mediating the Keap 1/*Nrf2* signaling pathway.

This study intends to (a) study the effect of lycopene on the growth performance, serum and liver antioxidant enzyme activity, and gene expression in the Keap 1/Nrf2 signaling pathway of Arbor Acres broilers by adding lycopene to the broilers' diets; (b) discuss the feasibility and appropriate contents of lycopene in the production of broilers; and (c) preliminarily explore the impact mechanism of the lycopene on production performance of broilers, to further develop and use the lycopene, and accumulate experience and theoretical basis for safe and reliable additives.

## Materials and Methods

### Chickens and Management

Newly hatched male Arbor Acres broiler chicks were purchased from Harbin Yinong Poultry Industry Co. Ltd. (Harbin, China) and raised in the Experimental Base of Northeast Agricultural University (Harbin, China). The protocols were approved by the Animal Care and Use Committee of the Animal Nutrition Institute, Northeast Agricultural University (NEAU-2018-0232). In addition, all animals were handled following the Ethics and Animal Welfare Committee of Heilongjiang Province, China. Chickens were reared in battery cages and exposed to 24 h of light with 15-W fluorescent lighting every day throughout the experiment. The average temperature for the first 3 days was 33°C, which reduced 2°C per week until it reached 23°C. The relative humidity in the room was controlled at 70% in the first 3 weeks, and then the relative humidity was maintained at 65%.

### Experimental Design and Diets

A total of 288 1-day-old male AA broilers were randomly divided into four groups with 6 replicates and 12 chickens for each replicate. The control group was fed the basal diet, while the treated groups were fed the basal diet with 10, 20, and 30 mg/kg lycopene in powder (lycopene concentration ≥96.0%; Nanjing Jingzhu Biotech. Co., Ltd., Nanjing, China). Feed and water were provided *ad libitum* for 42 days. Each group consisted of six replicates with 12 chickens per replicate. The composition of the basal experiment diets for birds is shown in Table 1. The trial was conducted in 2 phases, namely, a starter phase from 1 to 21 days and a grower phase from 22 to 42 days.

**Table 1:** Composition and nutrient content of the corn-soybean diets (air-dry basis).

**Ingredient**	**Starter diet** **(%, 1–21 days)**	**Grower diet** **(%, 22–42 days)**
Corn	57.85	62.3
Soybean meal	29	24
Corn protein meal	4.6	5
Cottonseed meal	2	2.5
Soybean oil	2.7	2.8
Dicalcium phosphate	1.9	1.65
Limestone	1.04	1
Salt	0.3	0.3
L-lysine HCl	0.09	0.03
Methionine	0.2	0.1
Choline chloride	0.1	0.1
Multi-vitamin^a^	0.02	0.02
Multi-mineral^b^	0.2	0.2
Total	100	100
**Nutrient level**		
Crude protein^c^	21.47	19.98
Available phosphorus^c^	0.45	0.37
Calcium^c^	1.01	0.93
Lysine^d^	1.16	1.00
Methionine and cysteine^d^	0.88	0.76
Threonine^d^	0.78	0.73
Tryptophan^d^	0.24	0.21
Metabolizable energy^c^, MJ/kg	12.53	12.76

a *The multi-vitamin provided the following per kilogram of diets: VA 8,000 IU; VD 4,000 IU; VE 12 mg; VK_3_ 1.6 mg; VB_1_ 2 mg; VB_2_ 6 mg; VB_6_ 3 mg; VB_12_ 0.012 mg; nicotinic acid 20 mg; folic acid 0.8 mg; biotin 0.04 mg; pantothenic acid 9 mg*.

b *The multi-mineral provided the following per kilogram of diets: Fe (as ferrous sulfate) 80 mg; Mn (as manganese sulfate) 100 mg; Zn (as zinc sulfate) 75 mg; Cu (as copper sulfate) 8 mg; I (as potassium iodide) 0.35 mg; Se (as sodium selenite) 0.15 mg*.

c *Analyzed content*.

d *Calculated value*.

### Data and Sample Collections

Body weight was recorded at 1, 21, and 42 days. Average daily gain, average daily feed intake, and feed conversion ratio were calculated by recording body weight and cumulative feed intake. At 21 and 42 days, respectively, the pre-slaughter weight was measured after 12 h of fasting, and 1 broiler with average body weight from each replicate was selected for blood sampling. Blood (10 ml) was collected with disposable vacuum blood collection tubes from the jugular vein and centrifuged at 3,000 × *g* for 10 min to obtain serum, which was divided into Eppendorf tubes and stored at a −20°C refrigerator for biochemical indicator testing. Ten grams of liver was collected into plastic packaging bags and stored until analyses at −20°C. Two grams of liver was collected into freezing tubes and stored at −80°C refrigerators for further assays.

### Antioxidant Enzyme Levels in Serum and Liver

The antioxidant parameters were assayed with an ultraviolet spectrophotometer (UV-2410PC, Shimadzu, Japan). The parameters evaluated in blood serum and liver included superoxide dismutase (SOD), total antioxidant capability (T-AOC), and glutathione peroxidase enzyme (GSH-Px) and malondialdehyde (MDA). The reagent kits were purchased from the Nanjing Jiancheng Biotechnology Co., Ltd. (Jiangsu, China), and the procedures were carried out following the manufacturer's instructions. Information on serum and liver biochemical indexes and the kits used in this study is shown in Table 2.

**Table 2:** Information on serum and liver biochemical indexes and the kits used.

**Items**	**Abbreviation**	**Kit No**.	**Coefficients of variation**	
			**Inter-assay**	**Intra-assay**
**Serum**				
Glutathione peroxidase enzyme	GSH-Px	A005-1-2	4.32%	4.36%
Superoxide dismutase	SOD	A001-3-2	4.56%	4.21%
Total antioxidant capability	T-AOC	A015-1-2	4.87%	4.63%
Malondialdehyde	MDA	A003-1-2	4.82%	4.58%
**Liver**				
Glutathione peroxidase enzyme	GSH-Px	A005-1-2	4.29%	4.56%
Superoxide dismutase	SOD	A001-3-2	4.36%	4.26%
Total antioxidant capability	T-AOC	A015-1-2	4.67%	4.33%
Malondialdehyde	MDA	A003-1-2	4.52%	4.68%

### Quantitative Real-Time PCR

Total RNA of the liver was isolated with TRIzol (Sigma, Saint Louis, MO) according to the procedure of the RNA extraction method. The quality of RNA was determined by 2% agarose gel electrophoresis, and total RNA concentration and purity (A260/A280 ratio) were ascertained with an ultra-micro spectrophotometer (NanoPhotometer, Implen, Germany). The total RNA from each liver was reverse transcribed into cDNA using a Prime Script™ RT reagent kit with gDNA Eraser (TaKaRa, Dalian, China). The obtained cDNA was used for qPCR using a TB Green™ Premix Ex Taq™ PCR kit (TaKaRa, Dalian, China) (20). The primer sequences are shown in Table 3, and the primers corresponding to the chicken gene sequence were synthesized by Sangon (Shanghai, China). Samples were disposed of with an ABI PRISM 7500 SDS thermal cycler (Applied Biosystems, Foster City, CA, USA). The following temperature program was used: one cycle at 95°C for 30 s, 40 cycles of 95°C for 5 s and 60°C for 34 s (20). The relative gene mRNA expression was calculated using the 2^−Δ*ΔCt*^ method and normalized to β-actin expression.

**Table 3:** Primers used for real-time PCR.

**Genes**	**Sequence (5^′^-3^′^)**	**Product size, bp**	**GenBank No**.
β-actin	F: TCAGGGTGTGATGGTTGGTATG	120 bp	NM_205518.1
	R: TGTTCAATGGGGTACTTCAGGG		
Keap1	F: GCTGCTGGAGTTCGCCTACAC	102 bp	XM_010728179.2
	R: CGCACCACGCTGTCGATCTG		
Nrf2	F: TGTGTGTGATTCAACCCGACT	143 bp	NM_205117.1
	R: TTAATGGAAGCCGCACCACT		
HO-1	F: TTGGCAAGAAGCATCCAGA	129 bp	NM_205344.1
	R: TCCATCTCAAGGGCATTCA		
NOQ1	F: CCCGAGTGCTTTGTCTACGAGATG	107 bp	NM_001277619.1
	R: ATCAGGTCAGCCGCTTCAATCTTC		
SOD2	F: TGCACTGAAATTCAATGGT	146 bp	NM_204211.1
	R: GTTTCTCCTTGAAGTTTGCG		
γ-GCS	F: ATGGCGTGGTTGGTGTTGCG	133 bp	NM 001004372.1
	R: TGAATTTGCGGGCGGACAGC		

*Keap1: kelch-like ECH-associated protein 1; Nrf2: NFE2-related factor-2; HO-1: heme oxygenase 1; SOD2: superoxide dismutase 2; NQO1: NAD(P)H quinone dehydrogenase 1; γ-GCS: γ-glutamylcysteine synthetase*.

### Statistical Analysis

All the data were tested for homogeneity of variance. The data were analyzed by one-way ANOVA and Tukey's multiple-range test in the Statistical Product and Service Solutions Software (SPSS 22.0; IBM-SPSS Inc., Chicago, IL, USA). The linear and quadratic effects of lycopene were assessed using regression analysis. Results were presented as the mean and the standard error of the mean (SEM). *p* < 0.05 was considered as a significant difference.

## Results

### Growth Performance

The growth performance data are summarized in Table 4. The ADG increased (*p* = 0.002 vs. *p* = 0.001) and the FCR decreased (*p* = 0.017 vs. *p* = 0.023) in groups treated with lycopene in the grower (22–42 days) and whole (1–42 days) phases, and the ADFI was quadratically affected (*p* = 0.043) by lycopene in the grower (22–42 days) phase compared with the control group.

**Table 4:** Effect of dietary lycopene levels on performance of AA broilers (1–42 days of age).

**Items**	**Lycopene levels (mg/kg)**				**SEM**	***p*** **-value**		
	**0 (Control)**	**10**	**20**	**30**		**ANOVA**	**Linear**	**Quadratic**
**Starter phase (1–21 days)**								
BW 21 days (g)	814.50	822.71	838.23	833.24	5.517	0.449	0.560	0.260
ADG (g)	36.77	37.16	37.88	37.63	0.260	0.466	0.558	0.274
ADFI (g)	42.41	41.07	42.00	42.32	0.373	0.593	0.199	0.379
FCR (feed:gain, g:g)	1.16	1.11	1.11	1.13	0.103	0.325	0.075	0.170
**Grower phase (22–42 days)**								
BW 42 days (g)	2,304.58^b^	2,456.00^a^	2,553.16^a^	2,538.36^a^	28.037	0.001	0.060	<0.001
ADG (g)	70.96^b^	77.78^a^	81.20^a^	81.66^a^	1.232	0.002	0.056	0.001
ADFI (g)	140.50	142.28	145.59	145.56	0.890	0.102	0.512	0.043
FCR (feed:gain, g:g)	1.99^a^	1.83^b^	1.79^b^	1.80^b^	0.265	0.017	0.044	0.006
**Whole phase (1–42 days)**								
ADG (g)	53.87^b^	57.47^a^	59.77^a^	59.41^a^	0.667	0.001	0.060	<0.001
ADFI (g)	91.46	91.68	93.80	93.94	0.509	0.157	0.912	0.070
FCR (feed:gain, g:g)	1.70^a^	1.60^b^	1.57^b^	1.59^b^	0.173	0.023	0.036	0.008

a, b *Means within a row with no common superscripts differ significantly (p < 0.05)*.

*BW, Body weight; ADG, Average daily gain; ADFI, Average daily feed intake; FCR, Feed conversion ratio*.

### Antioxidant Enzyme Activity in Serum

As summarized in Table 5, the MDA contents were lower (*p* ≤ 0.001) in the groups treated with lycopene than those in the control group, and the MDA contents in the 20 and 30 mg/kg lycopene groups were lower (*p* < 0.05) than those in the 10 mg/kg lycopene group at 21 days. The SOD contents were linearly affected (*p* = 0.035) by lycopene at 21 days. The GSH-Px, SOD, and T-AOC levels were higher (*p* = 0.014, *p* = 0.003, and *p* = 0.035, respectively) than those in the control group, and the T-AOC levels in 30 mg/kg lycopene were higher (*p* < 0.05) than those in the control group at 42 days.

**Table 5:** Effect of dietary lycopene on serum antioxidase of AA broilers.

**Items**	**Lycopene levels (mg/kg)**				**SEM**	***p*** **-value**		
	**0 (Control)**	**10**	**20**	**30**		**ANOVA**	**Linear**	**Quadratic**
**21 days**								
GSH-Px (U/ml)	750.48	813.07	867.24	855.93	33.81	0.636	0.228	0.425
SOD (U/mg prot)	187.04	191.01	211.70	209.31	4.73	0.148	0.035	0.108
T-AOC (U/ml)	0.58	0.54	0.61	0.61	0.03	0.794	0.523	0.755
MDA (nmol/mg prot)	3.21^a^	2.59^b^	1.74^c^	1.72^c^	0.15	<0.001	<0.001	<0.001
**42 days**								
GSH-Px (U/ml)	643.48^b^	748.99^a^	774.90^a^	789.28^a^	18.75	0.014	0.003	0.005
SOD (U/mg prot)	172.01^c^	193.59^bc^	216.59^ab^	227.54^a^	6.14	0.003	<0.001	0.001
T-AOC (U/ml)	0.46^b^	0.47^b^	0.52^ab^	0.58^a^	0.02	0.016	0.002	0.005
MDA (nmol/mg prot)	2.45	1.95	1.85	1.79	0.13	0.295	0.082	0.159

*GSH-Px, glutathione peroxidase enzyme; SOD, superoxide dismutase, T-AOC, total antioxidant capability, MDA, malondialdehyde*.

a, b, c *Means within a row with no common superscripts differ significantly (p < 0.05)*.

### Antioxidant Enzyme Content in Liver

The antioxidant enzyme contents in the liver are shown in Table 6. The GSH-Px and SOD contents in groups treated with lycopene were higher (*p* ≤ 0.001 vs. *p* ≤ 0.001) than those in the control group, and their contents were higher (*p* < 0.05) in the 30 mg/kg group than those in the 10 and 20 mg/kg groups at 21 days. The MDA contents were lower (*p* = 0.004) than those in the control group at 21 days. The GSH-Px contents were higher (*p* ≤ 0.001) in 30 mg/kg lycopene than those in the control and 20 mg/kg lycopene groups at 42 days. The MDA contents were lower (*p* = 0.002) in the groups treated with lycopene than those in the control group at 42 days.

**Table 6:** Effect of dietary lycopene on liver antioxidase of AA broilers.

**Items**	**Lycopene levels (mg/kg)**				**SEM**	***p*** **-value**		
	**0 (Control)**	**10**	**20**	**30**		**ANOVA**	**Linear**	**Quadratic**
**21 days**								
GSH-Px (U/mg prot)	1.25^c^	3.33^b^	2.72^b^	5.40^a^	0.38	<0.001	0.236	0.007
SOD (U/mg prot)	324.27^c^	450.74^b^	465.10^b^	534.79^a^	18.86	<0.001	<0.001	<0.001
T-AOC (U/mg prot)	55.45	62.89	63.98	63.83	1.79	0.282	0.102	0.149
MDA (nmol/mg prot)	1.08^a^	0.75^b^	0.70^b^	0.85^b^	0.04	0.004	0.066	0.001
**42 days**								
GSH-Px (U/mg prot)	10.76^b^	13.44^ab^	12.39^b^	14.43^a^	0.35	<0.001	<0.001	0.002
SOD (U/mg prot)	1,193.48	1,287.92	1,312.97	1,383.39	52.57	0.550	0.153	0.354
T-AOC (U/mg prot)	58.52	53.41	54.26	61.95	1.65	0.230	0.461	0.110
MDA (nmol/mg prot)	0.68^a^	0.48^b^	0.45^b^	0.53^b^	0.03	0.002	0.040	<0.001

*GSH-Px, glutathione peroxidase enzyme; SOD, superoxide dismutase, T-AOC, total antioxidant capability, MDA, malondialdehyde*.

a, b, c *Means within a row with no common superscripts differ significantly (p < 0.05)*.

### Quantitative Real-Time PCR of *Keap1-Nrf2* Pathway in Liver

**Figure 1:**
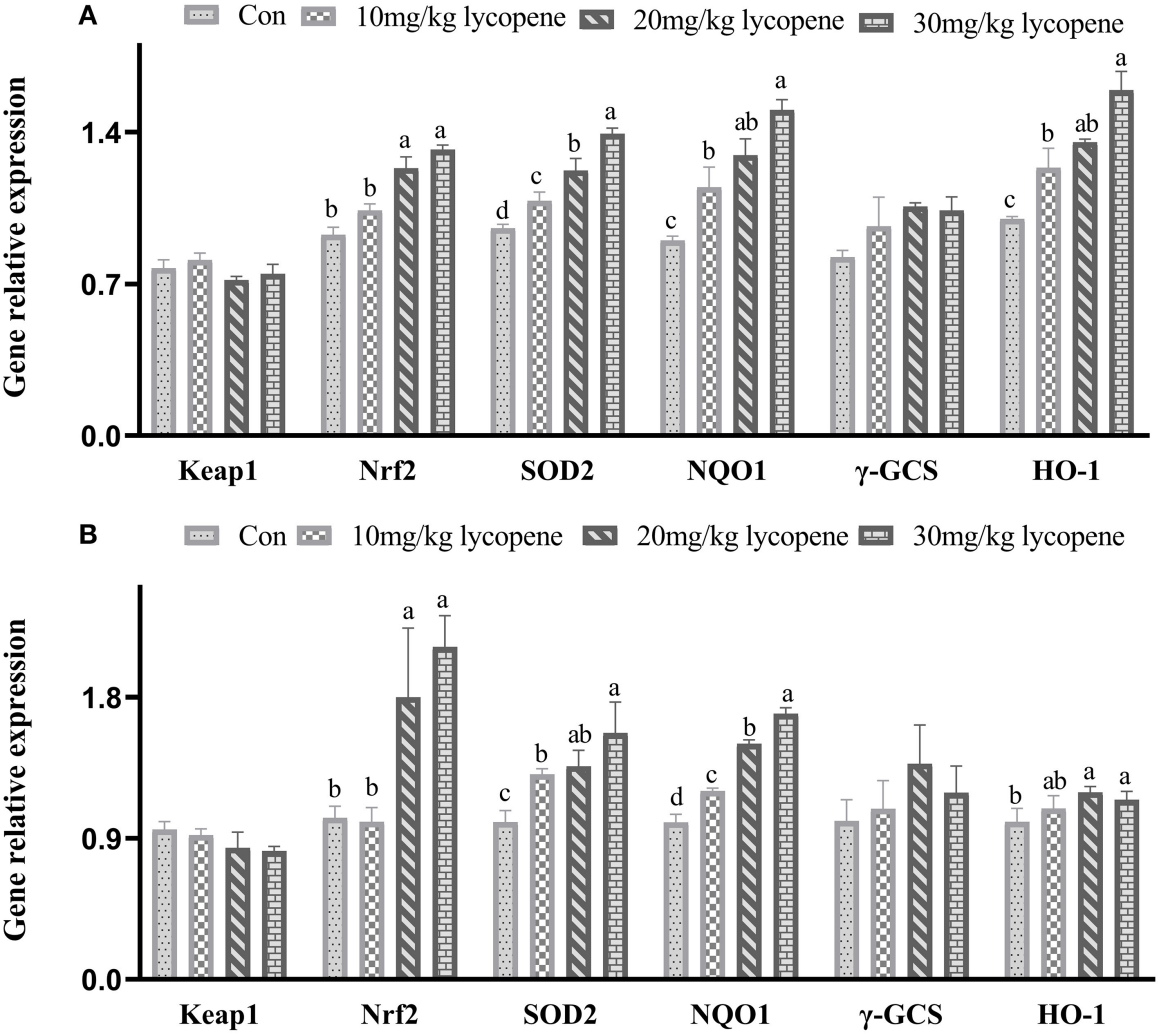
Effect of dietary lycopene levels on the *Keap1-Nrf2* regulated gene expression in the liver of AA broilers. **(A)** The data of *Keap1-Nrf2* regulated gene expression in the liver of AA broilers at 21 days in Con, 10, 20, and 30 mg/kg lycopene groups were as follows: *Keap* 1 gene: 0.77 ± 0.04, 0.81 ± 0.03, 0.72 ± 0.02, and 0.75 ± 0.04, respectively, *p* = 0.064; *Nrf2* gene: 0.93 ± 0.03^b^, 1.04 ± 0.03^b^, 1.23 ± 0.05^a^, and 1.32 ± 0.02^a^, respectively, *p* = 0.042; *SOD2* gene: 0.96 ± 0.02^d^, 1.08 ± 0.04^c^, 1.22 ± 0.05^b^, and 1.39 ± 0.02^a^, respectively, *p* = 0.021; *NQO1* gene: 0.90 ± 0.02^c^, 1.15 ± 0.09^b^, 1.29 ± 0.08^ab^, and 1.50 ± 0.04^a^, respectively, *p* = 0.035; γ*-CGS* gene: 0.82 ± 0.03, 0.97 ± 0.13, 1.06 ± 0.02, and 1.35 ± 0.5, respectively, *p* = 0.072; *HO-1* gene: 1.00 ± 0.01^c^, 1.24 ± 0.09^b^, 1.35 ± 0.01^ab^, and 1.59 ± 0.09^a^, respectively, *p* = 0.043. **(B)** The data of *Keap1-Nrf2* regulated gene expression in the liver of AA broilers at 42 days in Con, 10, 20, and 30 mg/kg lycopene groups were as follows: *Keap 1* gene: 0.96 ± 0.05, 0.92 ± 0.04, 0.84 ± 0.10, and 0.82 ± 0.03, respectively, *p* = 0.074; *Nrf2* gene: 1.03 ± 0.07^b^, 1.00 ± 0.09^b^, 1.80 ± 0.44^a^, and 2.12 ± 0.20^a^, respectively, *p* = 0.038; *SOD2* gene: 1.00 ± 0.07^c^, 1.31 ± 0.04^b^, 1.36 ± 0.10^ab^, and 1.57 ± 0.20^b^, respectively, *p* = 0.025; *NQO1* gene: 1.00 ± 0.05^d^, 1.20 ± 0.02^c^, 1.50 ± 0.02^b^, and 1.70 ± 0.04^a^, respectively, *p* = 0.034; γ*-CGS* gene: 1.00 ± 0.14, 1.09 ± 0.18, 1.37 ± 0.25, and 1.19 ± 0.17, respectively, *p* = 0.059; *HO-1* gene: 1.00 ± 0.08^b^, 1.09 ± 0.08^ab^, 1.19 ± 0.04^a^, and 1.15 ± 0.05^a^, respectively, *p* = 0.043.

As shown in Figure 1, the mRNA expression levels of *Nrf2, SOD 2, NQO1*, and *HO-1* genes were higher (21 days: *p* = 0.042, *p* = 0.021, *p* = 0.035, *p* = 0.043, respectively; 42 days: *p* = 0.038, *p* = 0.025, *p* = 0.034, *p* = 0.043, respectively) in the 20 and 30 mg/kg lycopene groups at 21 and 42 days. The mRNA expression levels of *Nrf2, NQO1*, and *HO-1* genes were higher (*p* <0.05) in the 30 mg/kg lycopene group than those in the 10 mg/kg lycopene group, and the mRNA expression levels of *SOD 2* genes were higher (*p* <0.05) in the 30 mg/kg lycopene group than those in the 10 and 20 mg/kg lycopene groups at 21 days. The mRNA expression levels of *Nrf2* and *SOD 2* genes were higher (*p* <0.05) in the 30 mg/kg lycopene group than those in the 10 mg/kg lycopene group, and the mRNA expression levels of *NQO1* genes were higher (*p* <0.05) in the 30 mg/kg lycopene group than those in the 10 and 20 mg/kg lycopene groups at 42 days.

## Discussion

Growth performance is an indispensable indicator of poultry growth status under different conditions (21), and the lycopene could improve chicken performance (22, 23). In the present study, the ADG increased, and the FCR decreased in groups treated with lycopene in the grower and whole phases, and the ADFI was quadratically affected by lycopene in the grower phase, which was consistent with Mezbani's study (22), who reported that chicken supplemented with 100 mg/kg lycopene from 21 to 42 days resulted in the increased body weight and decreased feed conversion ratio. However, no differences in growth performance were observed between the groups treated with lycopene and the control group during the starter phase (1–21 days), which might be that the low feed intake before 21 days reduced the lycopene intake. At the same time, the use of vaccines during this period will repeatedly cause immune stresses, and low lycopene dosage may not be sufficient to improve antioxidant capacity. Therefore, it could be considered that the amount of lycopene added can be increased during the early broilers' feeding process.

Antioxidant enzymes in poultry cooperative work to maintain the optimal redox balance *in vivo*, which regulates various processes, including cell signal transmission, gene expression, stress regulation, and homeostasis maintenance (12). The levels of T-AOC, SOD, GSH-PX, and MDA in liver and serum were usually used to evaluate the antioxidant potentials (24). In this study, the SOD, T-AOC, and GSH-Px levels of serum and liver were significantly increased and MDA levels were decreased, which coincide with the study of Sun et al. (25), who reported that diet with lycopene improved chick antioxidant enzyme activities of the liver, and declined liver MDA. Furthermore, detoxifying oxidative stress factors, GSH-PX and SOD enzyme reduced hydrogen peroxide and superoxide anions to non-toxic hydroxyl compounds (26). The improvement of antioxidant enzyme activity in serum and liver is beneficial to chicken, which contributes to the antioxidant properties of chicken, thereby increasing the daily weight gain (27). Previous studies have shown that supplements with antioxidants, such as selenium, vitamin C, and β-carotene, could improve broilers' antioxidant status and meat quality (27, 28). Similarly, it was proved that certain phytochemicals, such as flavonoids and polyphenols, improved the antioxidant capacity of broilers (29, 30). Lipid and protein oxidation have always been considered the main threats to meat quality except for microorganisms (31).

Nrf2, a key transcription factor, combines with the antioxidant response element (ARE), which activates target gene expression, and regulates the phase II detoxification enzymes and antioxidant enzymes (32). Numerous studies have reported that antioxidants exerted beneficial effects on the broiler by the *Keap1-Nrf2* signaling pathway (33, 34). Nevertheless, it was unclear whether lycopene activated the *Keap1-Nrf2* signaling pathway to promote the synthesis of the antioxidant enzymes in broilers. The present study showed that the liver *Nrf2* expression was upregulated in the 20 and 30 mg/kg lycopene groups, indicating that the *Nrf2* pathway was activated by lycopene. Under normal circumstances, *Nrf2* combines with Keap1 to form a complex that exists in the cytoplasm. *Nrf2* dissociates from Keap1, transfers to the nucleus under oxidative stress, and forms a heterodimer with Maf protein to initiate the transcription of downstream genes (35). However, *Keap1* expression was not affected by lycopene at the starter phase. Zhang (36) had demonstrated that the steady-state *Nrf2* expression increased significantly after tert-Butylhydroquinone (tBHQ) or sulforaphane treatment, and the tBHQ group significantly reduced the steady-state expression of *Keap1*. However, the sulforaphane group did not change the steady-state level of *Keap1*. *Nrf2* can bind to the ARE of the phase II genes and accelerates their transcription, including *NQO1, HO-1*, and *SOD2*, to maintain the redox balance (37). The results in this study showed that upregulated *Nrf2* regulated the antioxidant system to increase *NQO1* mRNA expression at the whole phase, indicating that its detoxification capacity was enhanced in the metabolic process. NQO1, a phase II metabolism enzyme, reduces quinone and nitrogen oxide compounds and alleviates oxidative damage (38). Similar to the present study results, Kaspar (39) reported that activating *Nrf2* can stimulate the expression of *NQO1*, which is positively correlated to *Nrf2*. HO-1 catalyzes heme to convert biliverdin, which has antioxidant and anti-inflammatory effects (40).

Additionally, SOD2 is a metalloenzyme composed of metal coenzymes and proteins, which can dismutase superoxide radicals into molecular oxygen and hydrogen peroxide (41). The present results showed that the gene expression levels of *SOD2* and *HO-1* in the liver were significantly increased after lycopene treatment. Several studies have shown that Nrf2 dissociates from Keap1 and into the nucleus, upregulating *Nrf2* expression and its downstream related genes, such as *HO-1, NQO1*, and *SOD* (42, 43). *Nrf2*-regulated is of great importance for antioxidant defense. Apart from that, whether changes in *Nrf2*-regulated gene expression in the liver activated by lycopene are affected by other signaling pathways remains to be further explored. The expression of *NQO1, HO-1*, and *SOD2* was upregulated, indicating the improved antioxidant potentials in broilers. Activating the *Keap1-Nrf2* signaling pathway by lycopene might contribute to redox status stability in broilers.

## Conclusions

The effects of lycopene on the growth performance, antioxidant enzyme activity in serum and liver, and gene expression in the Keap1/Nrf2 signaling pathway of Arbor Acres broilers in the different treatment groups were assessed. The average daily gain, feed conversion ratio, average daily feed intake, serum and liver glutathione peroxidase and superoxide dismutase contents, mRNAs of Nuclear Factor E2-related factor 2, superoxide dismutase 2, NAD(P)H quinone dehydrogenase 1, and heme oxygenase 1 gene expression levels were affected by lycopene in Arbor Acres broilers. The lycopene concentration of 30 mg/kg improved the growth performance, antioxidant enzyme activity in serum and liver, and gene expression in the *Keap1-Nrf2* signaling pathway of Arbor Acres broilers. In the early stages of broiler growth, the amount of lycopene added should be increased appropriately.

## Data Availability Statement

The original contributions presented in the study are included in the article/supplementary material, further inquiries can be directed to the corresponding author.

## Ethics Statement

The animal study was reviewed and approved by Animal Care and Use Committee of the Animal Nutrition Institute, Northeast Agricultural University (NEAU-2018-0232).

## Author Contributions

SW and HW prepared the manuscript and collected some data. YZ, HCu, JY, ML, HCh, LG, and TX collected the samples. LX was responsible for the design and direction of the experiment. All authors have read and agreed to the published version of the manuscript.

## Funding

This study was supported by the National Natural Science Foundation of China (32072749) and China Scholarship Council (CSC) National Public Graduate Program for Building Highly Qualified Universities.

## Conflict of Interest

The authors declare that the research was conducted in the absence of any commercial or financial relationships that could be construed as a potential conflict of interest.

## Publisher's Note

All claims expressed in this article are solely those of the authors and do not necessarily represent those of their affiliated organizations, or those of the publisher, the editors and the reviewers. Any product that may be evaluated in this article, or claim that may be made by its manufacturer, is not guaranteed or endorsed by the publisher.
